# Effects of Dexmedetomidine-Fentanyl Infusion on Blood Pressure and Heart Rate during Cardiac Surgery in Children

**DOI:** 10.1155/2010/869049

**Published:** 2010-08-19

**Authors:** Jyrson Guilherme Klamt, Walter Villela de Andrade Vicente, Luis Vicente Garcia, Cesar Augusto Ferreira

**Affiliations:** ^1^Department of Biomechanics, Medicine and Rehabilitation of the Locomotors System, Faculty of Medicine of Ribeirão Preto-USP, University of São Paulo, 14049-9005 Ribeirão Preto, SP, Brazil; ^2^Hospital das Clinicas da FMRP-USP, Serviço de Anestesia, Av. dos Bandeirantes 3900-Monte Alegre, 14048-900 Ribeirão Preto, SP, Brazil; ^3^Department of Surgery and Anatomy, Faculty of Medicine of Ribeirão Preto-USP, University of São Paulo, 14049-9005 Ribeirão Preto, SP, Brazil

## Abstract

*Background*. The purpose of this study was to access the effects of dexmedetomidine-fentanyl infusion on blood pressure (BP) and heart rate (HR) before surgical stimulation, on their changes to skin incision, and on isoflurane requirement during cardiac surgery in children. *Methods*. This study had a prospective, randomized, and open-label design. Thirty-two children aged 1 month to 10 years undergoing surgery for repair congenital heart disease (CHD) with CPB were randomly allocated into two groups: group MDZ received midazolam 0.2 mg·kg^−1^·h^−1^ and group DEX received dexmedetomidine 1 *μ*g·kg^−1^·h^−1^ during the first hour followed by half of these rates of infusions thereafter. Both group received fentanyl 10 *μ*g·kg^−1^, midazolam 0.2 mg·kg^−1^ and vecuronium 0.2 mg·kg^−1^ for induction. These same doses of fentanyl and vecuronium were infused during the first hour then reduced to half. The infusions started after induction and maintained until the end of surgery. Isoflurane was given briefly to control hyperdynamic response to skin incision and sternotomy. *Results*. In both groups, systolic blood pressure (sBP) and heart rate (HR) decreased significantly after one hour of infusion of the anesthetic solutions, but there were significantly less increase in diastolic blood pressure, sBP, and HR, and less patients required isoflurane supplementation to skin incision in the patients of the DEX group. *Discussion*. Dexmedetomidine infusion without a bolus appears to be an effective adjunct to fentanyl anesthesia in control of hemodynamic responses to surgery for repair of CHD in children.

## 1. Introduction

Midazolam (MDZ) is commonly combined with fentanyl for pediatric congenital heart disease (CHD) in order to provide hypnosis and deepening of the anesthetic level. This anesthetic regimen affords adequate analgesia and amnesia, and good hemodynamic stability, even in low cardiac reserve patients [1, 2].

Dexmedetomidine (DEX) is a highly specific and selective *α*_2_-adrenergic agonist with fast tissue distribution and short half-life capable of inducing controllable postoperative sedation and analgesia [3]. DEX shows superior sedation and similar respiratory and hemodynamic effects when compared to MDZ [4]. As an adjunct to general anesthesia, DEX decreases the anesthetic and opioid requirements in a variety of surgical procedures [3, 5], and contrary to MDZ, it does not increase the respiratory depressant effects of opioids [6]. In addition, DEX blunts the sympathetic-mediated hyperdynamic response to surgical stress [5] and attenuates the cardiovascular and neuroendocrine responses to surgery in pediatric cardiac surgery patients operated on under cardiopulmonary bypass (CPB) [7]. However, this last finding was observed in patients more than 1 year old, bearing relatively less complex CHD. 

This study compares the effects of the combination of DEX and fentanyl versus the combination of MDZ and fentanyl infusion on blood pressure (BP) and heart rate (HR) before surgical stimulation, their change to skin incision and sternotomy, and the isoflurane requirement to control hyperdinamic responses in infants and children undergoing cardiac surgery before CPB, including patients with low cardiac reserve. We hypothesized that DEX might be an effective substitute to MDZ during fentanyl anesthesia, based on DEX capacities to provide effective surgical sedation, to potentiate the opioid analgesia and to reduce the sympathetic tone, thus buffering the hemodynamic response to surgical stress.

## 2. Methods

After local approval and informed written consent, 32 consecutives patients undergoing elective open heart surgery with CPB were studied. Patients with previous cardiac surgery, metabolic, renal or hepatic disorders, prematurity, or left ventricular hypoplasic syndrome were excluded. Exclusion criteria also included cases where the central venous and arterial monitoring lines could not be cannulated within 50 minutes after induction, and those with single ventricle physiology. 

The patients were randomly allocated according to a computer generated random list into two groups: Fentanyl + MDZ infusion (group MDZ, *n* = 16), and fentanyl + DEX infusion (group DEX, *n* = 16). The study was not blind to the surgical and anesthetic teams. All patients had a peripheral IV line inserted preceding the anesthetic induction and received MDZ 0.2 mg·kg^−1^ at or right before being transported to the operating room (patients aged more than 6 months). All pre-operative vasoactive drugs infusion (dopamine, dobutamine, and PGF_2_) were maintained until the CPB was started. Anesthesia was induced with fentanyl (10 *μ*g·kg^−1^) and vecuronium (0.2 *μ*g·kg^−1^) to facilitate tracheal intubation. Noninvasive BP was measured every 2 min until an artery was cannulated for continuous BP measurement. All patients received promethazine (1.0 mg·kg^−1^), ranitidine (2.0 mg·kg^−1^), and methylprednisolone (60 mg·kg^−1^) right after the anesthetic induction.

After intubation and placement of nasopharyngeal, esophageal, and plantar surface temperature probes, and once adequate noninvasive BP (every two min), heart rate (HR), pulse oximetry (SpO_2_), end-tidal CO_2_ (E_T_CO_2_), and electrocardiogram tracings (ECG) were obtained, the anesthetic combination infusion was started. The MDZ group received an infusion of fentanyl (10 *μ*g·kg·^−1^h^−1^), MDZ (0.2 mg·kg^−1^·h^−1^), and vecuronium (0.2 mg·kg·^−1^h^−1^) for one hour, and the infusion rate was cut to half thereafter. In the DEX group, this drug replaced MDZ and was initially infused at 1.0 *μ*g·kg·^−1^h^−1^, and the infusion rate was managed just like in the other group. The drug infusion was mixed up in one syringe and was discontinued right before the patients were taken to the pediatric intensive care unit (PICU). Isoflurane (0.4–1%) was briefly employed to control 20–30% preinduction level rises in BP and or HR. 

The fractional inspiratory oxygen concentration (FiO_2_) was kept between 0.9-1.0 and the E_T_CO_2_ between 35-45 mmHg. Positive end-expiratory pressure of 2–5 cmH_2_O and inspiratory/expiration ratio (I : E) of 1 : 1.5–1 : 2 were used. The CPB was carried out at 2.5–3.0 L·min^−1^·m^2 (-1)^ on normothermia. The hematocrit was kept at 25% and the pH was managed by alpha-stat strategy. Mean arterial pressure was kept between 30 and 70 mmHg. Patients were cooled down to 26 to 32°C, at the surgeon's discretions.

Ringer solution was infused at 10 ml·kg^−1^·h^−1^ and 5 ml·kg^−1^ volume challenges in order to keep the CVP between 6–10 mmHg. After CPB, albumin 5% was employed instead. Dextrose (100 mg·kg^−1^) was given for hypoglycemia (≤70 mg·dl^−1^), but no dextrose containing solution was otherwise administered. Blood glucose levels and mixed venous hemoglobin oxygen saturation (SvO2) were measured every 10–20 minutes. Once CPB rewarming was initiated, a loading milrinone (MIL) dose (50 *μ*g·kg^−1^ over 10 minutes) was administered, followed by a 0.8 *μ*g·Kg^−1^·min^−1^ infusion of this drug in association with a 0.03–0.2 *μ*g·kg^−1^·h^−1^ epinephrine (EPI) infusion. EPI was reduced or even discontinued if BP, HR, central venous pressure (CVP), heart contractility, and SvO_2_ were adequate. If hypotension was presented during EPI and MIL infusion, norepinephrine was infused. Hypotension with normal HR and CVP was treated with IV norepinephrine (0.03–0.1 *μ*g·kg^−1^·min^−1^). Bradyardia associated with hypotension was managed with an epinephrine bolus (0.1–1.0 *μ*g·kg^−1^) or infusion (0.03–0.2 *μ*g·kg·min^−1^). Bradyardia without hypotension was treated with atropine (30 *μ*g·kg^−1^). These vasoactive drugs were used when BP and/or HR decreased to a value below the age-adjusted expected normal values [8], or decreased 30% from baseline levels (after the initial midazolam bolus administration). Phenylephrine (1–5 *μ*g·kg^−1^) was given to combat hypotension and hypoxemic crises in Tetralogy of Fallot patients. Tachycardia not associated with the surgical stimuli was controlled with 0.5–1.0 mg·kg^−1^ esmolol boluses. Sodium nitroprusside (SNP) (2 *μ*g·kg^−1^·min·^−1^) besides being infused in all patients during on CPB rewarming was used (2–8 *μ*g·kg·^−1^min·^−1^) to control systemic arterial hypertension during CPB. Transient BP falls secondary to surgical manipulation of the heart were not treated.

Preoperative cyanosis was defined as sustained low systemic arterial saturation (SpO_2 _≤ 90%) requiring nasal oxygen supplementation, during exercise, feeding, or crying in hospital before surgery. Preoperative heart failure was clinically determined by the pediatric cardiologist, on the basis of echography, heart catheterization data, or treatment with digitals and diuretics being provided within a week of the study. Pulmonary hypertension (PH) was determined on the preoperative echography or heart catheterization. 

Hemodynamic variables (HR, BP), temperatures (nasopharyngeal, esophageous, and plantar) were recorded in the following time periods: after the patient was sedated with MDZ (0.2 mg·kg^−1^) (M-baseline values), one hour after the anesthetic combination was initiated (1H), before skin incision (BS), immediately after skin incision (AS), after sternotomy (S), 10 minutes after of the sternotomy (S10), 3–5 minutes after protamine administration (P), and at the end of the operation (E). The intraoperative hemodynamic (BP and HR) changes present after one hour of the anesthetics infusion (during the period without surgical stimulation), immediately after skin incision, and isoflurane requirement to control increase 30% in BP and/or HR from baseline levels (hyperdinamic response) before CPB were the outcomes of primary interest. 

The sample size estimation and the statistical analysis were performed using GraphPad InStat 3.0 and GraphPad Prism 4, respectively, (GraphPad software, San Diego, CA, USA). To establish the sample, we took in consideration data of BP of previous studies (1,2,7) and finding from a performed pilot study as relevant clinical variable. The power calculation determined that the size of 14 in each group should be sufficient for 80% power to detect a difference of 20% in BP under a 5% significance level. The Friedman and Wilcoxon tests were used to analyze repeated measures from dependent variables and the Mann-Whitney *U*-test was employed to compare independent variables between the two groups at each moment, while the demographic parameters and adverse events were analyzed with the Fischer test. Most of the data are shown as mean ± SD. The level of significance was set at *P* < .05.

## 3. Results

A total of 37 patients were invited to participate, parents of five patients declined their children's participation, whereas the parents of the remaining patients approved their child participation with an informed consent. Two neonates from the MDZ group were excluded: one due to excessive time to insert central venous and arterial line and the other one because the planned surgical repair under CPB was changed over to a systemic-to-pulmonary shunt performed off bypass. Two neonates of the DEX group were also excluded from analysis because they required norepinephrine to maintain BP about 20 minute of induction: one 3 days old with IAoA receiving PGE_2_, dopamine, and milrinone infusions and the other 18 days old receiving PGE_2,_ milrinone, and furosemide infusions. DEX infusion was continued throughout the surgery in these two patients. Patient's characteristics, cardiac diagnoses, and CPB and aortic clamp durations are shown in Table 1. There was a tendency to lower weight and age in the DEX group as compared to MDZ group, but the difference did not reach statistical significance. Preoperative cyanosis was significantly more frequent in the DEX group, and more than 50% of the patients in both groups were in congestive heart failure. PH was present in about 40% of the patients in both groups.

**Figure 1:**
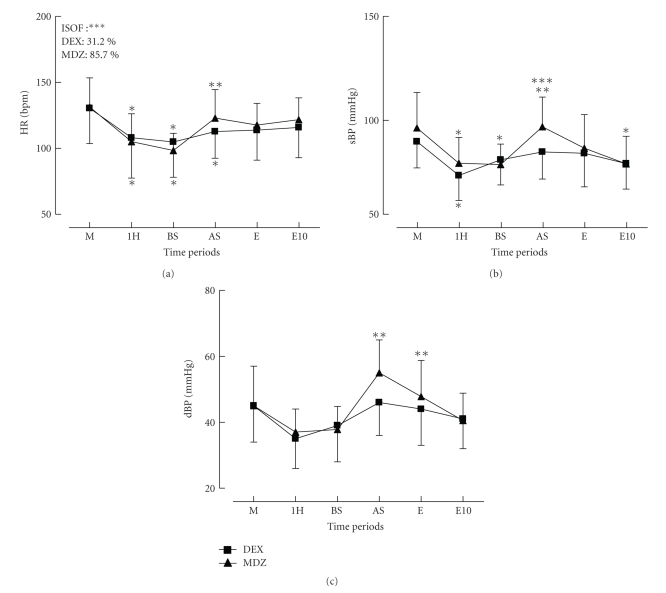
Effects of dexmedetomidine (DEX) on heart rate (HR) and diastolic (dBP) and systolic blood pressure (sBP) during cardiac surgery with CPB in children. M: after midazolan (MDZ) 0.2 mg·kg^−1^, 1H: after one hour of infusion of DEX (1 *μ*g·kg^−1^) or MDZ (0.2 mg·kg^−1^) associated with fentanyl (10 *μ*g·kg^−1^), BS: before skin incision, AS: after skin incision, E: after sternotomy, E10: 10 minutes after sternotomy. The box represents the number of patients that required isoflurane after skin incision. Data represent mean ± SD. *Significantly different from *M, from **BS, and ***between groups.

The hemodynamic variables are depicted in Figure 1. The baseline systolic blood pressure was significantly lower in the DEX group. HR and systolic BP decreased significantly after one hour of starting the test drug infusion in both groups. In the MDZ group, all hemodynamic variables increased significantly when the skin incision was performed. Although a similar trend was observed in the DEX group, it did not reach significance. Many more patients required isoflurane supplementation in the MDZ group than in the DEX group (85.7% versus 31.2%, *P* = .027). Adequate HR, BP, SvO_2_, and other respiratory variables were present in all patients from both groups on arrival at the PICU. 

In the DEX group, 3 patients came to the OR on PGE_2_ (Prostin) and milrinone, while another one was receiving dopamine. In the MDZ group, one patient was on PGE_2_ and milrinone, and another one was on dobutamine. Phenylephrine was used in one patient from the MDZ group and in two patients from the DEX group. In the DEX group, one patient was given atropine before CPB was initiated. 

## 4. Discussion

This randomized open-label design study evaluated the anesthetics effectiveness and safety of the fentanyl and DEX combination as compared to our routine technique in infants and children undergoing open heart surgery under CPB, which is based on fentanyl and MDZ infusion. Both anesthetics regimens provided effective anesthesia and could be easily supplemented by isoflurane to control hyperdynamic responses when necessary. The open label study was recommended because the two drugs (DEX and MDZ) have different pharmacological profile and their respective adverse effects require different management strategies. Both groups showed similar demographics, except for a tendency towards lower weight and age and higher incidence of cyanotic CHD in the DEX group, which baseline systolic BP was also lower. 

The fentanyl-MDZ-based protocol, aided by isoflurane, was adopted in our patient population, including infants and children, particularly for those with low cardiac reserve, on the basis that it has been shown to be effective and safe to providing analgesia and hypnosis with preservation of hemodynamic stability in children undergoing open heart surgery [2, 9, 10], although the DEX dose regimen was investigated mostly in nonpainful procedures such as magnetic resonance imaging [4, 11, 12], cardiac catheterization [13, 14], or broncoscopy [15]. Few studies have addressed the DEX dose regimen in children with CHD. The study by Mukhtar et al. [7] used a dosage similar to that recommended for normal adult patients (0.5 *μ*g·kg^−1^ bolus, followed by 0.5 *μ*g·kg^−1^·h^−1^ infusion) which was effective to attenuate the hemodynamic and neuroendocrine response to surgery, without any hemodynamic deleterious effects, in patients older than 1 year, with less complex CHD. On the other hand, Hammer et al. [16] showed that this dosage depress the sinus rate and atrioventricular nodal function in children undergoing electrophysiology study. Most commonly, DEX is administered as a bolus (0.5–1.0 *μ*g·kg^−1^) followed by an infusion rate of 0.2 to 2.0 *μ*g·kg^−1^·h^−1^. The loading dose may be followed by severe hypotension, bradycardia, or sinus arrest, especially during rapid loading, in young patients, with comorbid diseases and on medications capable of negative chronotropic effects (*β*-adrenergic antagonists, and digoxin) or with reduced blood volume [17, 18]. Since most patients of this study had these characteristics, the loading dose was omitted in order to avoid rapid hemodynamic changes. The 1 *μ*g·kg^−1^·h^−1^ DEX infusion initiated immediately after induction should not had compromised the sedation as it was gradually replacing the MDZ (0.2 mg·kg^−1^) sedation. This strategy of gradual substitution of MDZ by DEX is frequently used in intensive care unit [19, 20]. The 0.5 *μ*g·kg^−1^·h^−1^ rate has been shown to be very effective in providing sedation and analgesia for mechanical ventilation even without any previous bolus [21].

The primary outcomes of this study were the hemodynamic effects of the combined fentanyl and DEX infusion as compared to our routine anesthetic technique (fentanyl and MDZ infusion). In both experimental groups, HR and systolic BP significantly decreased during the one hour period of administration before surgical stimulation was started, but a hyperdynamic response to skin incision was significant only in the MDZ group, whose patients required more isoflurane supplementation as well. The potentiation of the opioid analgesia is in agreement with the well-known DEX opioid sparing effect [3]. Nevertheless, two patients who came to the OR on PGE_2_ and milrinone infusion experienced BP below acceptable levels and had to be given norepinephrine infusion. Our results are also consistent with other studies showing that DEX provides effective sedation and analgesia associated with hemodynamic stability and blunts sympathetic responses during critical moments of surgery such as skin incision and sternotomy, but can lead to life-threatening hypotension and bradycardia requiring prompt vasoactive support [2, 3, 7].

It is well known that fentanyl has a rapid action onset and generates minimal hemodynamic changes even when large doses are given in pediatric patients with very low cardiac reserve. However, when in combination with benzodiazepines, circulatory depression requiring volume challenge and, in some cases, inotropic support may occur [1, 2, 9, 19]. Rivenes et al. [1] demonstrated that the combination of fentanyl and MDZ depressed the HR, BP, and cardiac index, notwithstanding the myocardial contractility remained the same. For this reason, in case of hemodynamic disturbances caused by induction (fentanyl + MDZ), we start a epinephrine, and/or norepinephrine infusion, if the effect a single dose of single dose of atropine, epinephrine or phenylephrine and a volume challenge were not sufficient. As stated above, only two patients, both in the DEX group, required intervention to control hypotension, and this could be due to an interaction of DEX with vasodilators (milrinone and PGE_2_). Whether the fentanyl and DEX combination had compromised the cardiac output (CO) in the same intensity as the fentanyl and MDZ combination could not be proven in our study, despite a similar SvO2 was present at the time recorded. It is known, however, that a DEX high infusion rate of 0.5 *μ*g·kg^−1^·h^−1^ can reduce the CO [22]. Conversely, DEX has been used as total intravenous anesthesia at doses as high as 10 *μ*g·kg^−1^·h^−1^ without inducing hypotension or severe bradycardia [15, 23]. Recently, Mason et al [12] have shown that a DEX high dose (a bolus of 2-3 *μ*g·kg^−1^ over 10 minutes, infusion of 1.5–3.0 *μ*g·kg^−1^·h^−1^) in children undergoing MRI produces bradycardia in 16% of these patients, but the mean arterial pressure and oxygen saturation remained at normal range, and no adverse squeals were observed and no specific treatment was required. Nevertheless, children with cardiac disease were excluded from the study and no opioid was administered. To date, this is the first report of the combination of anesthetics doses of fentanyl and effective sedative dose of DEX in infants and children undergoing open heart surgery will CPB. Mukhtar et al. study [7] involved children older than one year with less complex CHD and used lower doses of fentanyl. The two anesthetics regimens herein compared showed similar effects after CPB discontinuation. In fact, all patients of the two groups arrived at the PICU with adequate hemodynamic, respiratory function and lactate levels. Nowadays, similarly to Easley and Tobias [24] recommendation, for children older than one year and not receiving vasoactive support, we start the DEX infusion soon after induction and in infant only after obtaining central venous and arterial lines.

The potential for hypotension and systemic vasodilatation due to DEX sympatholitic action carries great concern in children with cyanotic CHD, as it may increase the right-to-left shunt thus worsening hypoxemia. Despite of having significant more patients with cyanotic CHD, the DEX group showed no significant changes in pulse oximetry as compared to MDZ group. Possibly, fentanyl and a high FiO_2_ might have reduced the pulmonary vascular resistance and offset this deleterious effect. Caution should also be exercised in patients with fixed CO such as severe aortic stenosis, since vasodilatation can lead to low coronary and cerebral perfusion [25]. Due to the scarce experience with DEX in pediatric patients undergoing open heart surgery or in critical state treatment, there are yet no data concerning the DEX effects on the balance between pulmonary and systemic vascular resistance in the single ventricle physiology; we, therefore, excluded children undergoing Norwood, Glenn, and Fontan surgery from our study.

In conclusion, the combination of fentanyl-DEX infusion provided effective anesthesia for pediatric patients undergoing cardiac surgery, when compared to a control group that received fentanyl-midazolam infusion anesthesia. In addition, the fentanyl-DEX group hyperdynamic response to surgical stimuli was blunted. However, a worrisome hypotensive response may ensue and need prompt treatment, particularly in patients already receiving vasodilators. 

## Figures and Tables

**Table 1:** Demographic, congenital heart disease (CHD), cardiopulmonary bypass (CPB), and aortic cross clamp time, and preoperative incidence of cyanosis (C), heart failure (H), and pulmonary hypertension (P) data. Values represent individual values and mean (m) ± SD. *Statistical significance, *P* < .05.

	DEX						MDZ					
Pt	Ag	We	CHD	C*	H	P	Ag	We	CHD	C	H	P
1	84	32	ASD			+	0.7	3.3	DORV, VSD	+	+	+
2	3	3.2	AVC, PDA		+	+	12	6.2	ASD, PDA		+	+
3	4	3.9	AVC		+	+	7	4.3	DORV, VSD			
4	3	5.6	TOF	+			4	4	ASD, VSD			+
5	2	3.6	TAr	+	+	+	36	14	VSD			
6	12	8.3	TOF	+			47	14	PVS			
7	4	5.3	ASD, VSD		+	+	12	8	TOF	+		
8	7	6.3	AVC,VSD		+	+	2	3.8	VSD		+	
9	0.1	3.3	TGV				8	8.2	AVC		+	+
10	6	6.5	AVC	+	+		24	9.2	AVC, ASD, VSD		+	+
11	7	7.2	PVS	+	+		108	28	SVAoS		+	
12	0.2	2.9	TGA, PDA	+			1.4	4.3	TGV, VSD	+	+	+
13	0.6	3.3	DORV,VSD	+	+		71	30	ASD			
14	19	10	TOF	+			7	6	TOF	+	+	

m ±	10.9	7.2					24.3	12.2				
SD	20.9	7.2					20.5	8.4				

Gender; M : F			6 : 8						8 : 6			
CPB; min			101.9 ± 41.5						117.6 ± 44.2			
Aortic cross clamp; min			71.5 ± 39.7						76.1 ± 26.7			

Pt: patients; Ag: age (months), We: weight (kg); ASD: atrial septal defect, AVC: atrioventricular canal defect, DORV: double-outlet of right ventricule, IAoA: interrupted aortic arch, PDA: patent ductus arteriosus, PVS: pulmonary valve stenosis, SVAoS: subvalvar aortic stenosis, TAr: truncus arteriosus, TGV: transposition of the great vessels, TOF: tetralogy of Fallot, and VSD: ventricular septal defect.
